# Supplementary material for: The genome sequence of the Orange Footman, Eilema sororcula (Hufnagel, 1766)
Source: Wellcome Open Res. 2023 Jun 28;8:282. [Version 1] doi: 10.12688/wellcomeopenres.19626.1 (PMC10905117; doi:10.12688/wellcomeopenres.19626.1)
Supplement: Peer review history

# Reviewer report: Reviewer response for version 1

**DOI:** 10.21956/wellcomeopenres.21742.r72054

**Published:** 2024-02-28

**Authors:** Marta Vila

**Affiliations:** 1 Universidade da Coruna, A Coruña, Galicia, Spain

This is a fine "genome note". I hope the following comments will help the authors to improve the manuscript.

 BACKGROUND

 1. I think many readers will benefit from a sentence where the authors clarify why they use the name Eilema sororcula and not Wittia sororcula (e.g. Bayarsaikhan et al. 2016).

 2. Please indicate that future studies should complement the present work by sequencing a female -preferrably from the same population- so that information of the W chromosome is produced.

 3. Please indicate if the karyotype of the species was known (if so, does your inferred number of chromosomes match?) or if you are the first researchers to indicate the euploid number of this species. In any case, the manuscript would benefit from briefly discussing how 2n = 30 fits within the available karyotypes for Eilema/Arctiinae. 

 References:[1]

Are sufficient details of methods and materials provided to allow replication by others?

Yes

Is the rationale for creating the dataset(s) clearly described?

Yes

Are the datasets clearly presented in a useable and accessible format?

Yes

Are the protocols appropriate and is the work technically sound?

Yes

Reviewer Expertise:

Genetics, Lepidoptera

I confirm that I have read this submission and believe that I have an appropriate level of expertise to confirm that it is of an acceptable scientific standard, however I have significant reservations, as outlined above.

## References

[1] : Review of the Eilema group of lichen moths from South Korea, with description of one new species (Lepidoptera: Erebidae: Arctiinae: Lithosiini). *Journal of Forestry Research* .2016;27(2) : 10.1007/s11676-015-0207-9 407-417 10.1007/s11676-015-0207-9 38220694

# Reviewer report: Reviewer response for version 1

**DOI:** 10.21956/wellcomeopenres.21742.r72064

**Published:** 2024-02-27

**Authors:** Juan Galarza

**Affiliations:** 1 University of Oulu, Oulu, Finland

The study presents the chromosomally complete genome sequence of the Orange Footman moth ( *Eilema sororcula)* based on a male specimen collected from Wytham Woods, Oxfordshire, UK. This species historically had a local distribution in the south and east of the UK until the late 20th century, but recent observations indicate a northward spread and a significant increase in both distribution and abundance. The species has also been recorded across Europe and eastwards to Korea and China.

*E. sororcula* is known for its preference for mature woodlands, where its larvae feed on algae and lichens growing on trees. 

 The chromosomally complete genome sequence was obtained from one male specimen using Pacific Biosciences single-molecule HiFi long reads and 10X Genomics read clouds, with additional scaffolding through chromosome conformation Hi-C data. Manual assembly curation addressed missing joins, mis-joins, and haplotypic duplications. The final assembly comprises 40 sequence scaffolds with a total length of 729.4 Mb, and 99.94% of the sequence assigned to 30 chromosomal-level scaffolds.

 The study emphasizes that the genome sequence of *E. sororcula* will contribute to molecular adaptation studies related to lichen-feeding. It adds to the growing dataset of genomic resources for understanding lepidopteran biology. The comprehensive methods employed in the study, including sequencing, assembly, and curation, contribute to the reliability of the genomic data presented. This resource has the potential to further scientific investigations into the ecological and molecular aspects of *E. sororcula* and enhance our understanding of its expanding distribution and ecological adaptations.

 The methods employed in this study are appropriate and technically sound. The researchers have followed established protocols, used recognized tools, and provided comprehensive documentation throughout the experimental procedures. The paper adheres to the standards of genomics research, and the detailed methods section facilitates potential replication by other researchers.

Are sufficient details of methods and materials provided to allow replication by others?

Yes

Is the rationale for creating the dataset(s) clearly described?

Yes

Are the datasets clearly presented in a useable and accessible format?

Yes

Are the protocols appropriate and is the work technically sound?

Yes

Reviewer Expertise:

Bioinformatics, Molecular Ecology

I confirm that I have read this submission and believe that I have an appropriate level of expertise to confirm that it is of an acceptable scientific standard.

# Reviewer report: Reviewer response for version 1

**DOI:** 10.21956/wellcomeopenres.21742.r61541

**Published:** 2023-06-30

**Authors:** Michael Hiller

**Affiliations:** 1 LOEWE-Centre for Translational Biodiversity Genomics (TBG), Senckenberg Nature Research Society, Frankfurt Am Main, Germany

This data note reports a reference-quality genome assembly of *Eilema sororcula*, together with a gene annotation produced by Ensembl. The generated data and methods used are according to the latest standards. The resulting assembly has a very high quality.

 I have 3 minor comments:

1. typo: collected from| Wytham Woods
2. "Most (99.94%) of the assembly sequence was assigned to 30 chromosomal-level scaffolds" - Although I understand that these sentences are generated by filling placeholders with the real values, I think the word 'most' does not capture that essentially the entire assembly (99.94%) is in chrom-level scaffolds. I would suggest to write "The vast majority of the assembly" or something similar, here and in the abstract.
3. "While not fully phased, the assembly deposited is of one haplotype. Contigs corresponding to the second haplotype have also been deposited. " - I don't understand this point. The authors likely produced a primary assembly (and provide alternative contigs in the 'alt assembly'), although this is not precisely mentioned in the methods (HiFiasm has different assembly modes). If so, the primary is likely a mix of both haplotypes, and not 'one haplotype' as stated. This should please be clarified.

Are sufficient details of methods and materials provided to allow replication by others?

Yes

Is the rationale for creating the dataset(s) clearly described?

Yes

Are the datasets clearly presented in a useable and accessible format?

Yes

Are the protocols appropriate and is the work technically sound?

Yes

Reviewer Expertise:

Genome assembly / comparative genomics

I confirm that I have read this submission and believe that I have an appropriate level of expertise to confirm that it is of an acceptable scientific standard.
